# Supplementary material for: Tomosyns attenuate SNARE assembly and synaptic depression by binding to VAMP2-containing template complexes
Source: Nat Commun. 2024 Mar 26;15:2652. doi: 10.1038/s41467-024-46828-1 (PMC10965968; doi:10.1038/s41467-024-46828-1)
Supplement: Supplementary file 1 — Supplementary Information [file 41467_2024_46828_MOESM1_ESM.pdf]

Supplementary Information

Tomosyns attenuate SNARE assembly and synaptic depression by binding to VAMP2-containing template complexes

Marieke Meijer[1, 5], Miriam Öttl[2, 5], Jie Yang[3, 5], Aygul Subkhangulova[2], Avinash Kumar[3], Zicheng Feng[3], Torben W. van Voorst[2], Alexander J. Groffen[1], Jan R.T. van Weering[1], Yongli Zhang[3, 4, 6, *] and Matthijs Verhage[1, 2, 6, *]

[1] Department of Human Genetics, Center for Neurogenomics and Cognitive Research, Amsterdam University Medical Center, 1081HV Amsterdam, The Netherlands
[2] Department of Functional Genomics, Center for Neurogenomics and Cognitive Research, Vrije Universiteit Amsterdam, 1081HV Amsterdam, The Netherlands
[3] Department of Cell Biology, Yale School of Medicine, New Haven, CT 06511, USA
[4] Department of Molecular Biophysics and Biochemistry, Yale University, New Haven, CT 06511, USA
[5] These authors contributed equally: Marieke Meijer, Miriam Öttl, Jie Yang
[6] These authors jointly supervised this work: Yongli Zhang, Matthijs Verhage

*Correspondence: m.meijer@vu.nl (M.M.), jie.yang.jy546@yale.edu (J.Y.), Yongli.zhang@yale.edu (Y.Z), m.verhage@vu.nl (M.V.)

Supplementary Fig. 1

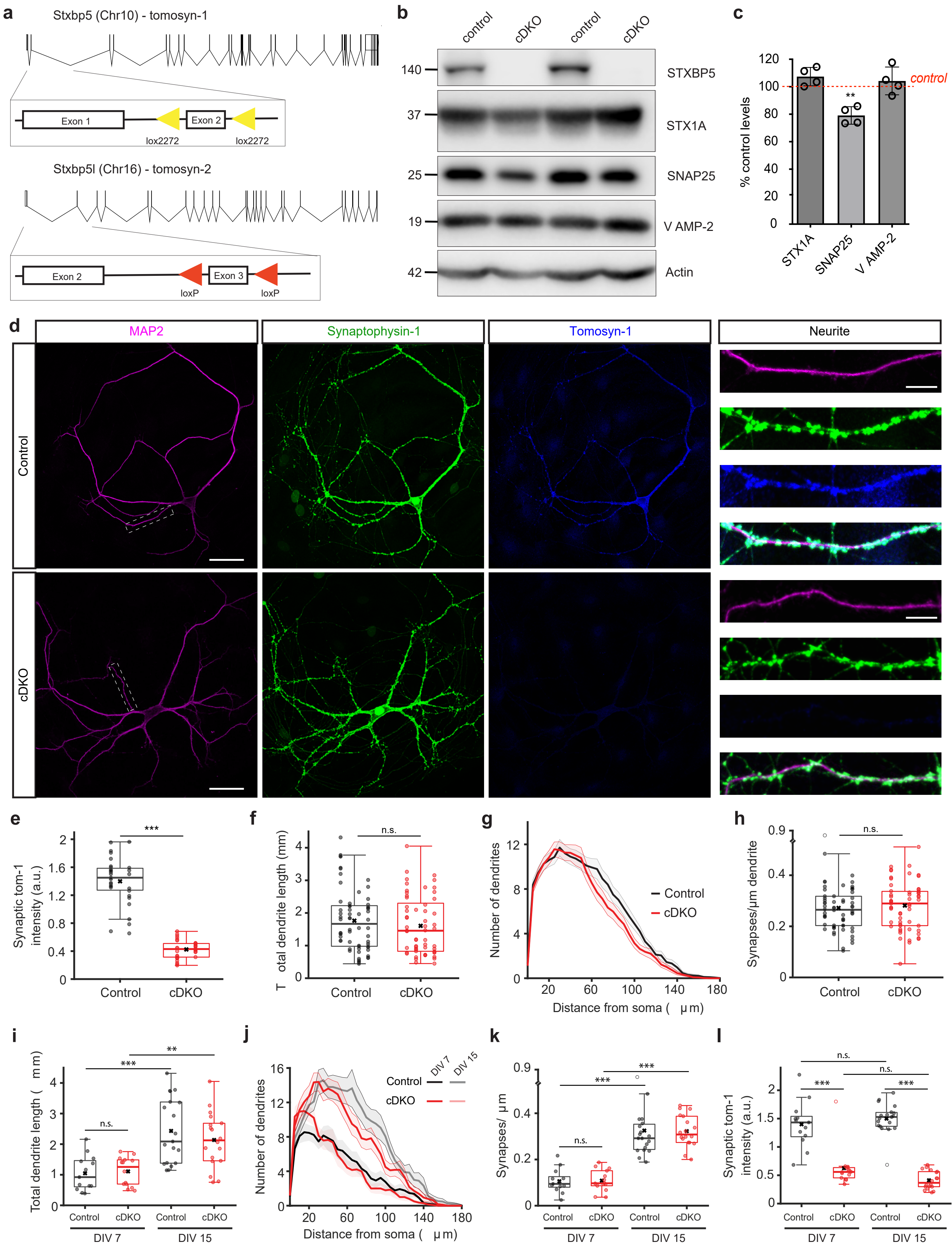

Supplementary Figure 1. **A novel conditional KO mouse for both tomosyn paralogs. a** Structures of the tomosyn-1 and tomosyn-2 genes. Zoom-ins to early exons show floxed regions around exon 2 of tomosyn-1 and exon 3 of tomosyn-2. **b** Example Western blot showing loss of tomosyn-1 (STXBP5) and levels of synaptic SNAREs in cDKO high-density neuronal cultures. Numbers on the left indicate approximate molecular weight of the detected bands in kDa. **c** Quantification of synaptic SNAREs expression from Western blots exemplified in **b**. cDKO levels were normalized to control levels in the corresponding culture after normalizing to actin. N = 4 independent cultures. Open circles denote the average of 3 technical replicates per culture. Bars show means of biological replicates ± SD. Data were analyzed using one sample t-test. $^{**}p<.01$. **d** Example images of autaptic hippocampal neurons immunostained for MAP2 as a dendrite marker, synaptophysin-1 as a synapse marker, and tomosyn-1 (scale bar = 50 µm). Dashed boxes in MAP2 images correspond to dendrite zoom-ins on the right. Dendrite zoom-ins show synaptic localization of tomosyn-1 and loss of expression in cDKO neurons (scale bar = 10 µm). **e-h** Quantifications from images shown in **d. e** Loss of tomosyn expression in synapses was confirmed by measuring the intensity of the tomosyn-1 signal within synaptophysin-1 puncta in cDKO (n = 30/2) and control (n = 31/2) neurons; $^{***}p<.0001$. **f** Total dendritic length was determined by tracing of the MAP2 signal. Control n = 62/4, cDKO n = 57/4; $p=0.295$. **g** Sholl plot showing similar dendritic complexity of both genotypes. **h** Synapse density was determined by the number of synaptophysin-1-positive puncta within one µm dendrite. Control n = 62/4, cDKO n = 57/4; $p=0.2035$. **i-k** Morphological analysis from immunostainings at day in vitro (DIV) 7 and 15 shows similar morphology of both groups throughout development. DIV7: control n = 13/1, cDKO n = 14/1; DIV15: control n = 19/1, cDKO n = 18/1. **i** Total neurite length derived from MAP2 tracing; $p=0.7628$ (DIV7: WT vs cDKO), $^{***}p=0.0002$ (WT: DIV7 vs DIV15), $p=0.3685$ (DIV15: WT vs cDKO), $^{***}p=0.0005$ (cDKO: DIV7 vs DIV15). **j** Sholl plot of dendrites. **k** Synapse density was determined from synaptophysin-1-positive puncta within one µm dendrite; $p=0.8865$ (DIV7: WT vs cDKO), $^{***}p<0.0001$ (WT: DIV7 vs DIV15), $p=0.2869$ (DIV15: WT vs cDKO), $^{***}p<0.0001$ (cDKO: DIV7 vs DIV15). **l** Signal intensity of tomosyn-1 in synaptophysin-positive puncta confirms loss of tomosyn at both DIV7 and DIV15; $^{***}p<0.0001$ (DIV7: WT vs cDKO), $p=0.1895$ (WT: DIV7 vs DIV15), $^{***}p<0.0001$ (DIV15: WT vs cDKO), $p=0.0215$ (cDKO: DIV7 vs DIV15).

N = cells/independent cultures, unless stated otherwise. In **e**, **f**, **h**, **I**, **k** and **l**, boxplots display median (center), upper and lower quartiles (box bounds) and whiskers to the last datapoint within 1.5x interquartile range. In **g** and **j**, data are presented as mean ± SEM. A one-way ANOVA tested the significance of adding experimental group as a predictor, see Supplementary Table 1. When applicable, p-value thresholds (*<0.05; **<0.01;***<0.001) were adjusted with a Bonferroni correction (α/number of tests). Abbreviations: n.s. (not significant); DIV (day in vitro). Source data are provided as a Source Data file.

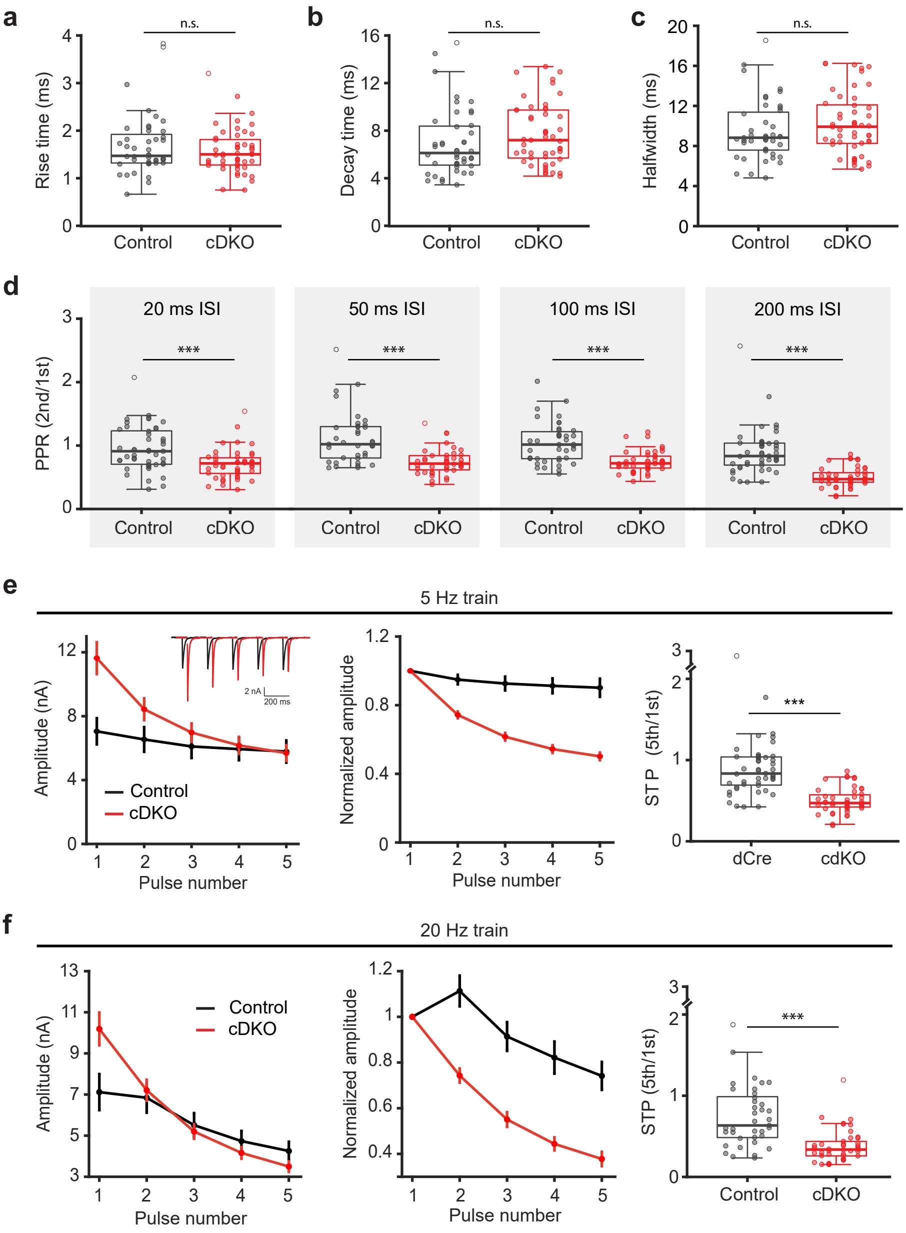

Supplementary Figure 2. **Normal EPSC kinetics and more quantifications of release probability and short-term plasticity. a-c** Analysis of the kinetics of single EPSCs. Control n = 44/6, cDKO n = 49/6. a 20 – 80% rise time; *p*=0.7124. **b** 100 – 50% decay time; p=0.0602. **c** EPSC width at half maximum; *p*=0.0907. **d** Paired-pulse ratios at different inter-pulse intervals. Same data as in Fig. 1 g. 20 ms IPI: control n = 45/6, cDKO n = 47/6; ****p*<0.0001. 50 ms IPI: control n = 37/6, cDKO n = 42/6; ****p*<0.0001. 100 ms IPI: control n = 39/6, cDKO n = 44/6; ****p*<0.0001. 200 ms IPI: control n = 47/6, cDKO n = 47/6; ****p*<0.0001. **e** Same analysis as in Fig. 1 h-k but for a 5 Hz train of 5 pulses. The rundown of the absolute (left) and normalized (center) amplitude were plotted and the ratio of the fifth over the first amplitude was calculated (right) to quantify short-term plasticity. Control n = 39/6, cDKO n =44/6; ****p*<0.0001. **f** Same as in e but for a 20 Hz train. Control n = 39/6, cDKO n = 42/6; ****p*<0.0001.

N = cells/independent cultures. In **a-f**, boxplots display median (center), upper and lower quartiles (box bounds) and whiskers to the last datapoint within 1.5x interquartile range. In left and center plots in **e** and **f**, data are presented as mean ± SEM. A one-way ANOVA tested the significance of adding experimental group as a predictor, see Supplementary Table 1. Abbreviations: n.s. (not significant). Source data are provided as a Source Data file.

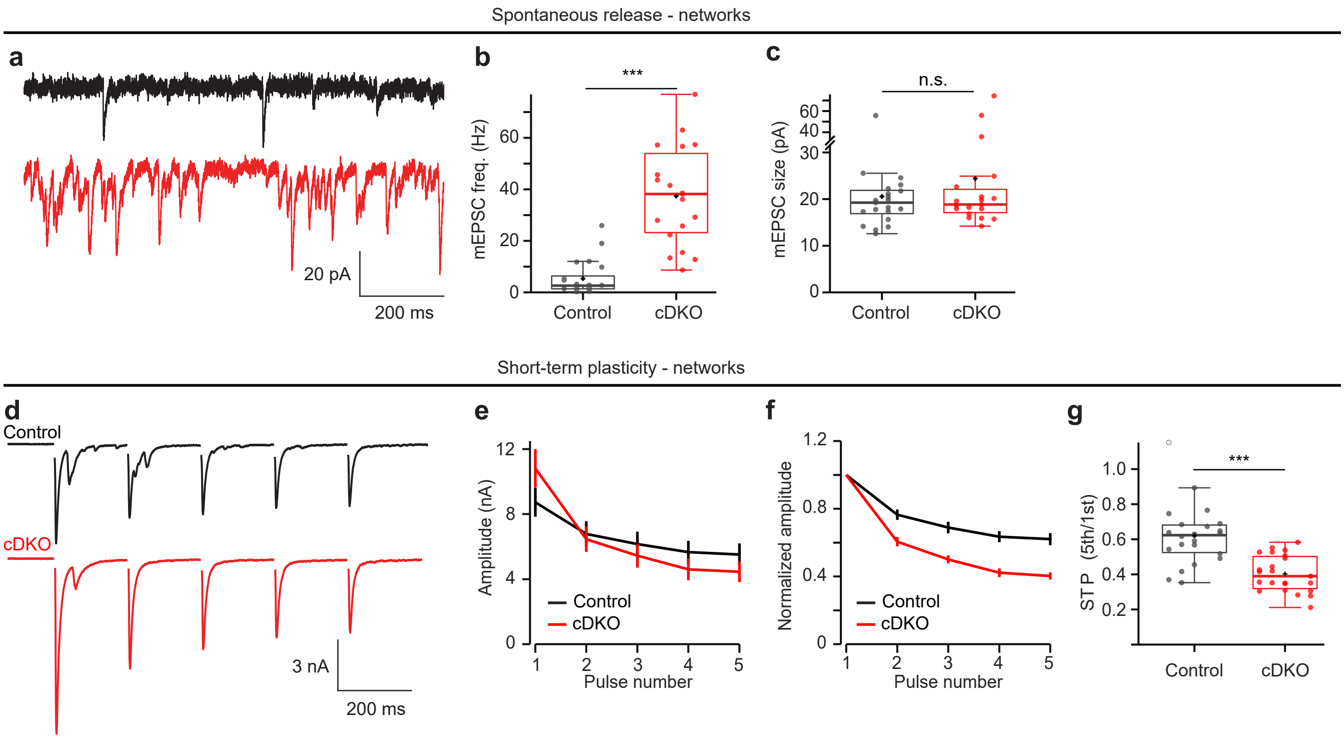

Supplemental Figure 3. **Tomosyn cDKO neurons in micro-networks enhances the release probability of spontaneous and evoked release. a-c** Analysis of spontaneous vesicle release in micro-networks of hippocampal neurons. Control n = 21/4, cDKO n = 19/4. **a** Example traces of miniature EPSCs (mEPSCs). **b** The frequency of mEPSCs; ****p*<0.0001. **c** mEPSC amplitude; *p*=0.2802. **d-g** Analysis of evoked synaptic transmission in micro-networks of hippocampal neurons. Control n = 23/5, cDKO n = 23/5. **d** Example traces of EPSCs evoked by local field stimulation (5 pulses at 5 Hz). **e** Amplitudes of EPSCs. **f** Amplitudes of EPSCs normalized to the first pulse. **g** STP quantified by the ratio of the fifth pulse over the first pulse; ****p*<0.0001.

N = cells/independent cultures. In **b**, **c** and **g**, boxplots display median (center), upper and lower quartiles (box bounds) and whiskers to the last datapoint within 1.5x interquartile range. In **e** and **f**, data are presented as mean ± SEM. A one-way ANOVA tested the significance of adding experimental group as a predictor, see Supplementary Table 1. Abbreviations: n.s. (not significant). Source data are provided as a Source Data file.

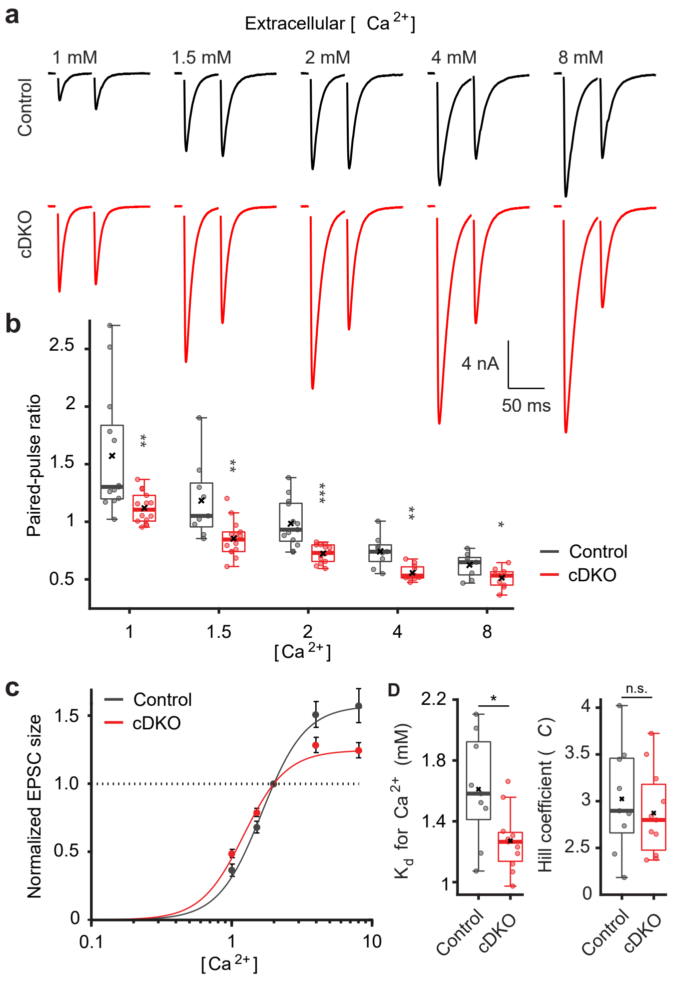

Supplementary Fig. 4. **Tomosyns increase the calcium affinity of the release machinery. a-b** Autaptic hippocampal neurons were stimulated with paired pulses of 50 ms intervals at different extracellular calcium concentrations. Control n = 9 – 13/4, cDKO n = 10 – 14/4. **a** Example traces. **b** The paired-pulse ratio (PPR) was calculated by dividing the amplitude of the second pulse by the amplitude of the first pulse. 1mM: control n = 13/4, cDKO n = 14/4, ***p*=0.0052; 1.5mM: control n = 9/4, cDKO n = 14/4, ***p*=0.0086; 2mM: control n = 13/4, cDKO n = 14/4, ****p*=0.0002; 4mM: control n = 9/4, cDKO n = 11/4, ***p*=0.0028; 8mM: control n = 9/4, cDKO n = 10/4, **p*=0.0288. **c** Concentration-response curves of the 1st EPSC amplitudes from data in a-b, normalized to responses in standard 2 mM [Ca^{2+}]. **d** A Hill function was fitted to the data shown in **c** from which the dissociation constant (Kd) for calcium (left) and the Hill coefficient (right) were calculated. Control n = 9/4, cDKO n = 11/4; **p*=0.0193 (Kd); *p*=0.7792 (Hill coefficient).

N = cells/independent cultures. In **b** and **d**, boxplots display median (center), upper and lower quartiles (box bounds) and whiskers to the last datapoint within 1.5x interquartile range. In **c**, data are presented as mean ± SEM. A one-way ANOVA tested the significance of adding experimental group as a predictor, see Supplementary Table 1. Abbreviations: n.s. (not significant). Source data are provided as a Source Data file.

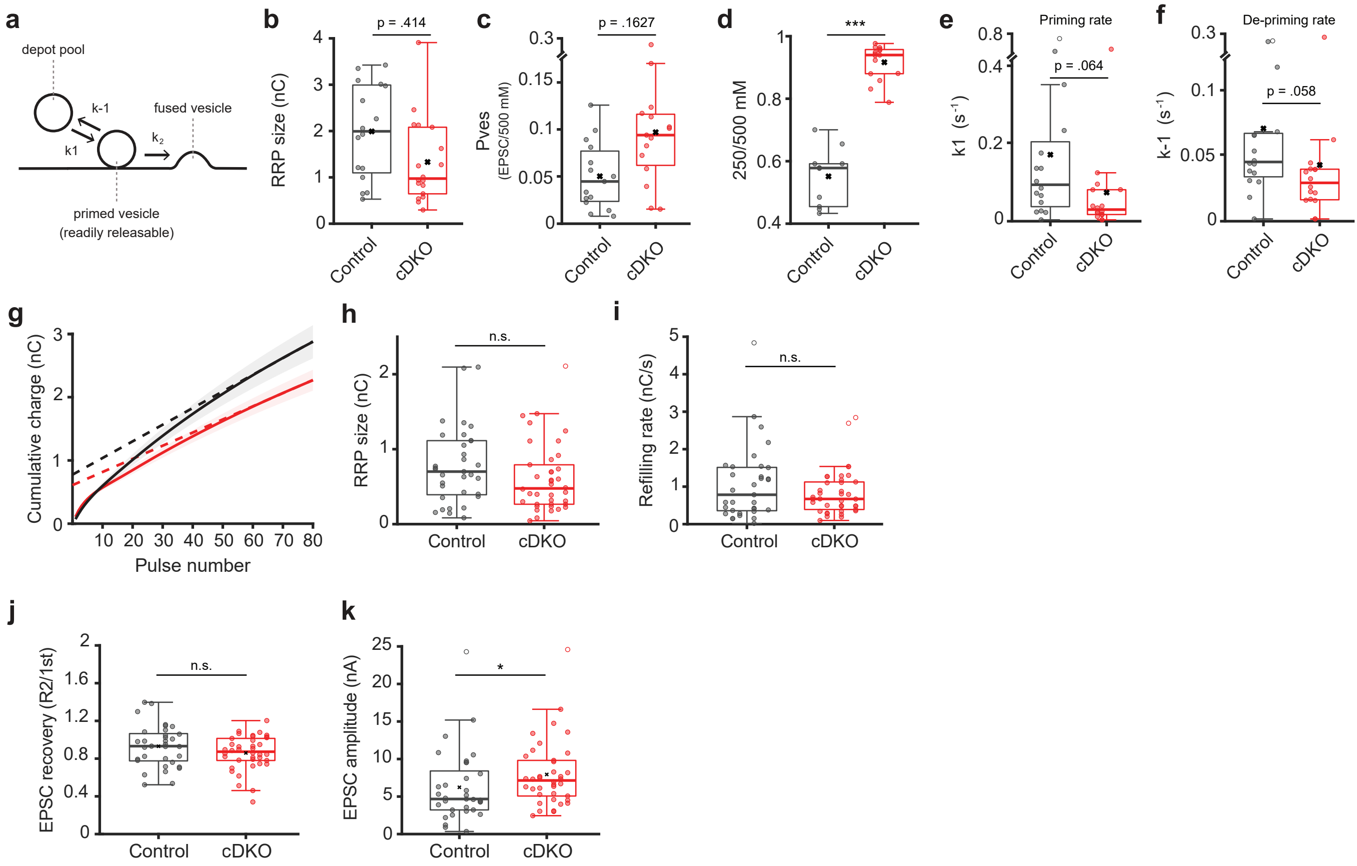

Supplementary Figure 5. **Fitting of an energy barrier model yields similar results as manual analysis of sucrose traces. a** Illustration of the minimal vesicle state model. Vesicles first start out in a depot pool of vesicles from which they transition to a primed state at a priming rate k1. De-priming occurs at a rate of k-1. Primed vesicles will fuse with a rate k2. **b** RRP size as estimated from the fitted data. Control n = 16/6, cDKO n = 18/6. **c** Pves calculated as the ratio of the charge released by a single EPSC to the fitted RRP. Control n = 15/6, cDKO n = 15/6. **d** The fraction of the RRP released by the submaximal sucrose concentration as calculated from the fitted data. Control n = 10/6, cDKO n = 15/6; ****p*<0.0001. **e** Priming and **f** de-priming rates during the 500 mM sucrose application, derived from the fitting. Control n = 16/6, cDKO n = 16/6. **g** Neurons were stimulated with 80 action potentials at 40 Hz (see Fig. 2g). The cumulative charge released during the train was plotted and a line was drawn through the last 20 pulses to back-extrapolate to the y-intercept, which marks the estimate for the RRP (readily releasable pool) size. h RRP size as calculated by back-extrapolation shown in **g**. Control n = 33/6, cDKO n = 38/6; *p*=0.1090. **i** The refilling rate of vesicles during the pulse is estimated from the slope of the back-extrapolation line shown in **g**. Control n = 33/6, cDKO n = 38/6; *p*=0.1419. **j** The amplitude of recovery pulse R2 (see Fig. 2 g) was divided by the first amplitude of the train. Control n = 31/6, cDKO n = 35/6; *p*=0.1933. **k** The absolute amplitude of recovery pulse R2. Control n = 31/6, cDKO n = 35/6; **p*=0.0316.
N = cells/independent cultures. In **b-f** and **h-k**, boxplots display median (center), upper and lower quartiles (box bounds) and whiskers to the last datapoint within 1.5x interquartile range. In **g**, data are presented as mean ± SEM (shaded area). A one-way ANOVA tested the significance of adding experimental group as a predictor, see Supplementary Table 1. Source data are provided as a Source Data file.

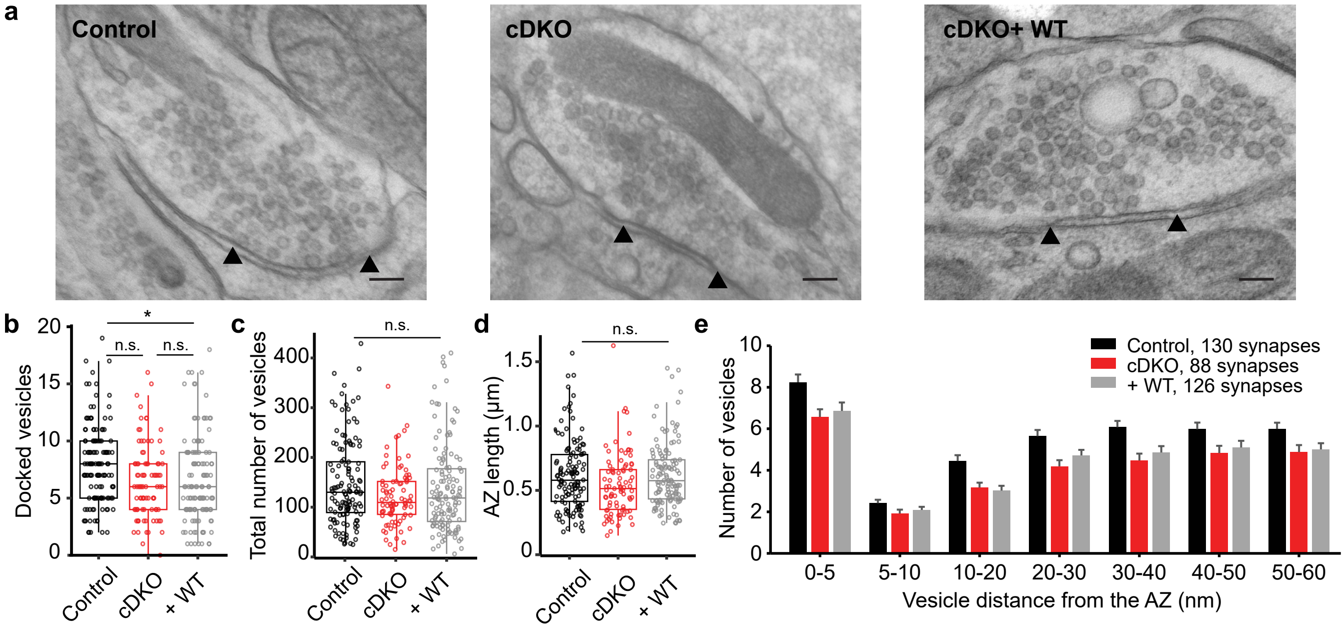

Supplementary Figure 6. **Loss of tomosyns does not increase vesicle docking.**

a-e High-pressure freeze electron microscopy was performed on micro-networks of control (n = 132 synapses / 4 sapphires), cDKO (n = 89 synapses/ 4 sapphires), and cDKO neurons expressing tomosyn-1m (+WT; n = 128 synapses/ 4 sapphires). **a** Example images. Edges of the active zone region on the presynaptic membrane are indicated by arrowheads. Scale bar = 100 nm. **b** No significant difference between control and cDKO synapses is detected, but the number of docked vesicles is reduced in + WT synapses compared to control. p=0.0424 (control vs cDKO); *p=0.0153 (control vs +WT), p=0.804 (cDKO vs +WT). **c** The total number of synaptic vesicles (p=0.3097) and **d** the length of the active zone (p=0.2044) in the cross section is normal in all groups. **e** The distribution of vesicles proximal to the active zone is normal in all groups.

A one-way ANOVA tested the significance of adding experimental group as a predictor, see Supplementary Table 1. Abbreviations: n.s. (not significant). Source data are provided as a Source Data file.

Supplementary Fig. 7

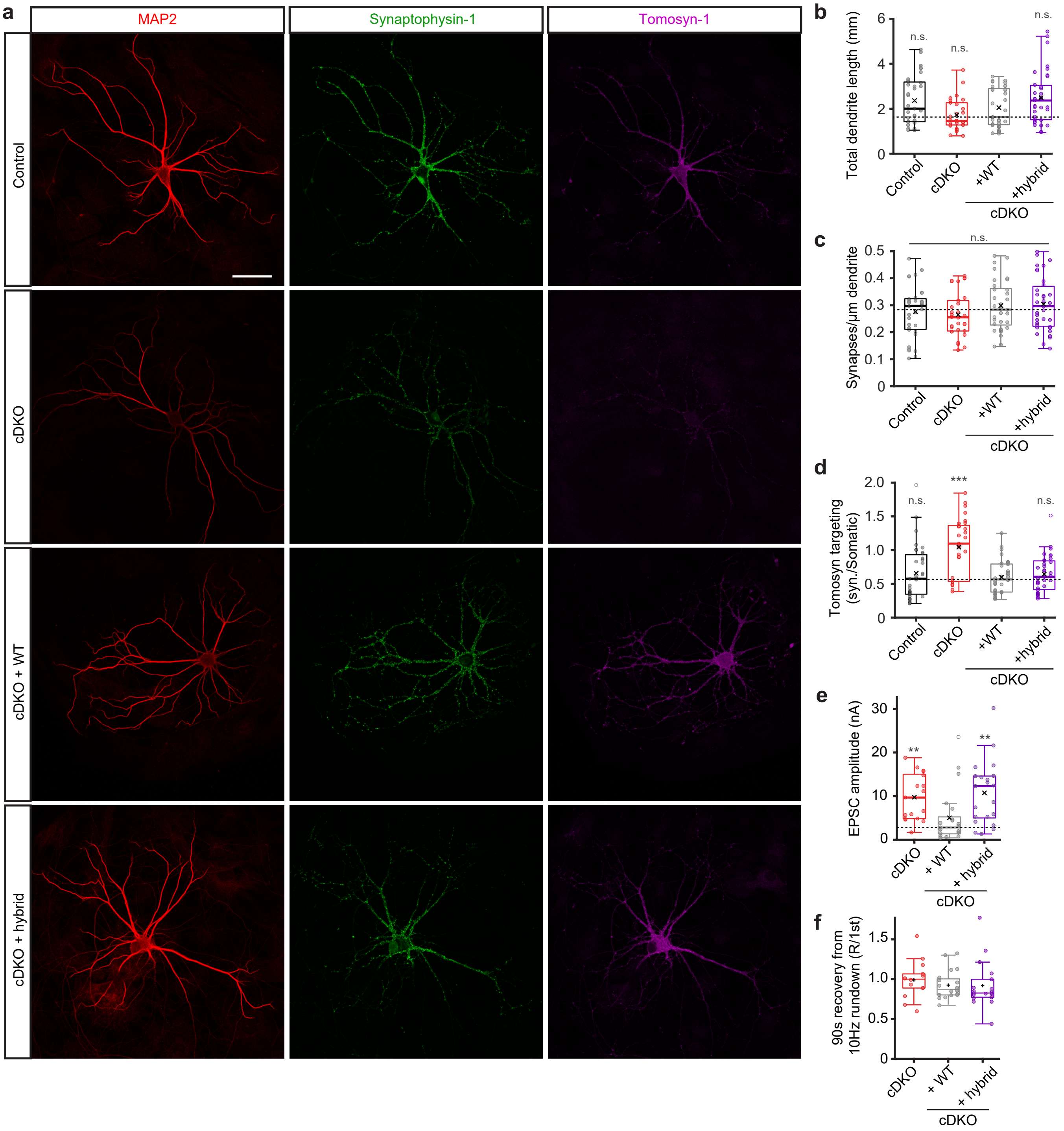

Supplementary Figure 7. **The tomosyn-VAMP2 hybrid is properly targeted to synapses. a-d** Morphological analysis of neurons infected with wild-type tomosyn or hybrid tomosyn (see Fig. 3). Control n = 35/3, cDKO n = 30/3, + WT n = 33/3, + Hybrid n = 35/3. **a** Example images of autaptic hippocampal neurons immunostained for MAP2, synaptophysin-1, and tomosyn-1. Scale bar = 50 µm. **b** Total dendrite length as derived from the MAP2 mask; *p*=0.0622 (control vs +WT); *p*=0.0826 (cDKO vs +WT), *p*=0.0354 (+hybrid vs +WT). **c** Synapse density as derived from the synaptophysin-1-positive puncta within the MAP2 mask; *p*=0.2991 (all groups). **d** The ratio of the tomosyn-1 signal intensity in synapses to the signal in the soma was calculated to demonstrate synaptic targeting; *p*=0.2934 (control vs +WT); ***p<0.0001 (cDKO vs +WT), *p*=0.3730 (+hybrid vs +WT). **e** Absolute EPSC amplitudes. cDKO n = 17/4, + WT n = 21/4, + Hybrid n = 21/4; **p=0.00137 (cDKO vs +WT), **p=0.00147 (+hybrid vs +WT). **f** EPSC amplitude measured 90 seconds after STP protocol (5 pulses at 10Hz) normalized to initial EPSC amplitude (1st pulse 10Hz train). cDKO n = 14/4, + WT n = 20/4, + Hybrid n = 18/4.

N = cells/independent cultures. In **b-f**, boxplots display median (center), upper and lower quartiles (box bounds) and whiskers to the last datapoint within 1.5x interquartile range. A one-way ANOVA tested the significance of adding experimental group as a predictor, see Supplementary Table 1. For post-hoc comparison to + WT group, p-value thresholds (*<0.05; **<0.01;***<0.001) were adjusted with a Bonferroni correction (α/number of tests). Grey asterisks show comparison to + WT group. Abbreviations: n.s. (not significant). Source data are provided as a Source Data file.

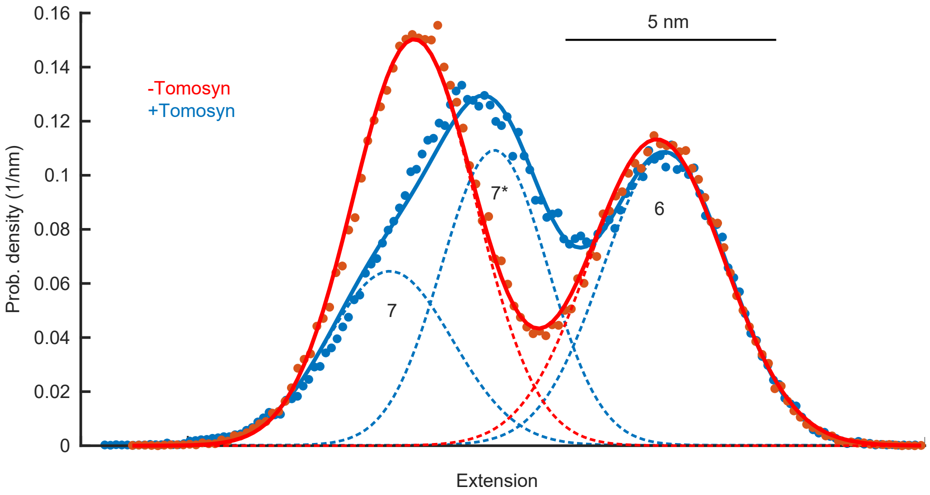

Supplementary Figure 8. **The probability density functions (PDFs) of the time-dependent extensions at constant forces revealed an intermediate state induced by the tomosyn SNARE motif (state 7*).** The PDFs can be well fitted by a sum of two Gaussian functions in the absence of the tomosyn SNARE motif and three Gaussian functions when present. The three peaks represent the template complex (state 7), the tomosyn-bound template complex (state 7*), and the Munc18-1-bound open syntaxin (Fig. 5 c).

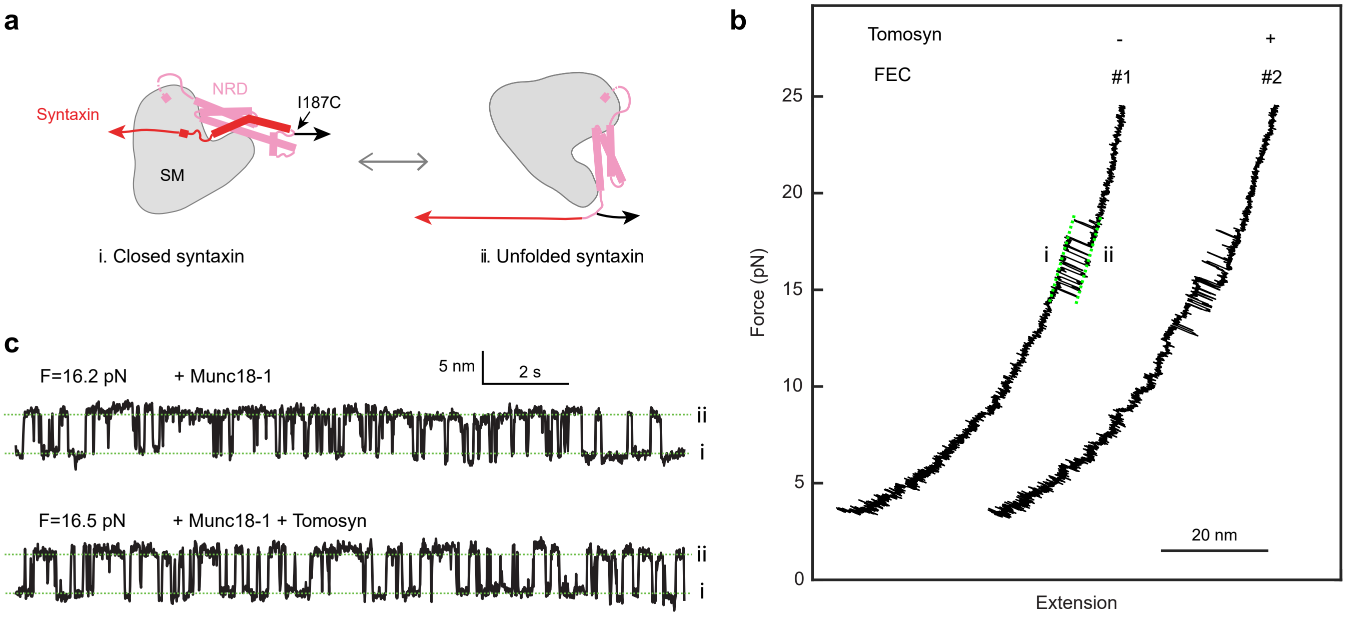

Supplementary Figure 9. **The tomosyn SNARE motif does not affect the folding of Munc18-1-bound closed syntaxin-1. a** Schematic diagrams of Munc18-1-bound closed syntaxin (i) and unfolded SNARE motif (ii). Note that the syntaxin-1 molecule was pulled from its C-terminus and I187C to which a DNA handle was attached. **b** Force-extension curves obtained by pulling Munc18-1 bound syntaxin-1 in the absence or presence of 2 µM tomosyn SNARE motif in the solution. **c** Extension-time trajectories at constant forces showing reversible unfolding and refolding of the Munc18-1-bound syntaxin.

Abbreviations: N-terminal regulatory domain (NRD).

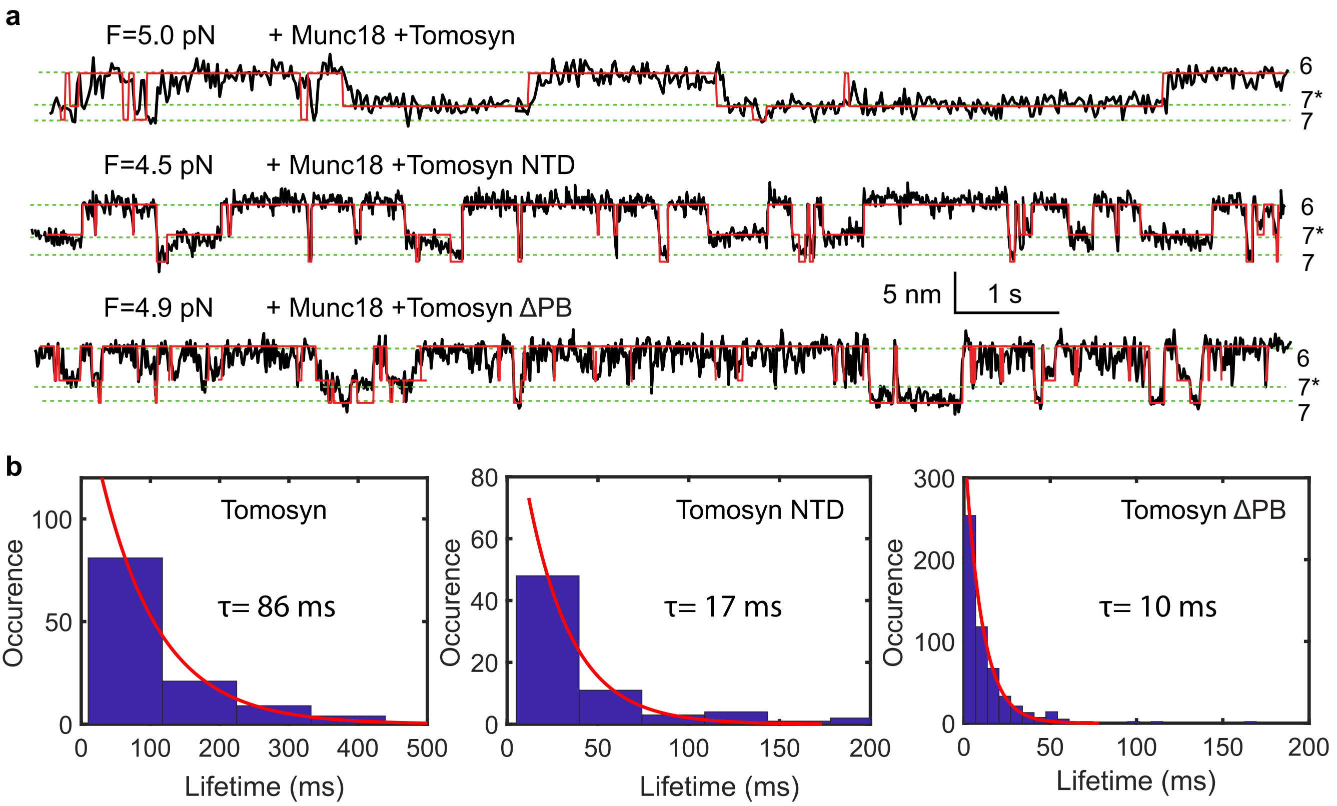

Supplementary Figure 10. **Reduced lifetimes of the tomosyn-bound template complex state due to tomosyn truncation.** **a** Closed-up views of the extension trajectories (black) containing the tomosyn-bound template complex state shown in Fig. 6a. **b** Histograms of the dwell time of the tomosyn-bound template complex. The whole trajectories span 40 s-90 s and were first analyzed by three-state hidden-Markov modeling. Then, the idealized state transitions (red traces in a) were derived by the Viterbi algorithm. Finally, the states corresponding to the tomosyn-bound template complex state (state 7*) were extracted and their dwell times were calculated for the histogram distributions. The distributions were fit with a single exponential function (red curves) to derive the average lifetimes (τ) indicated.

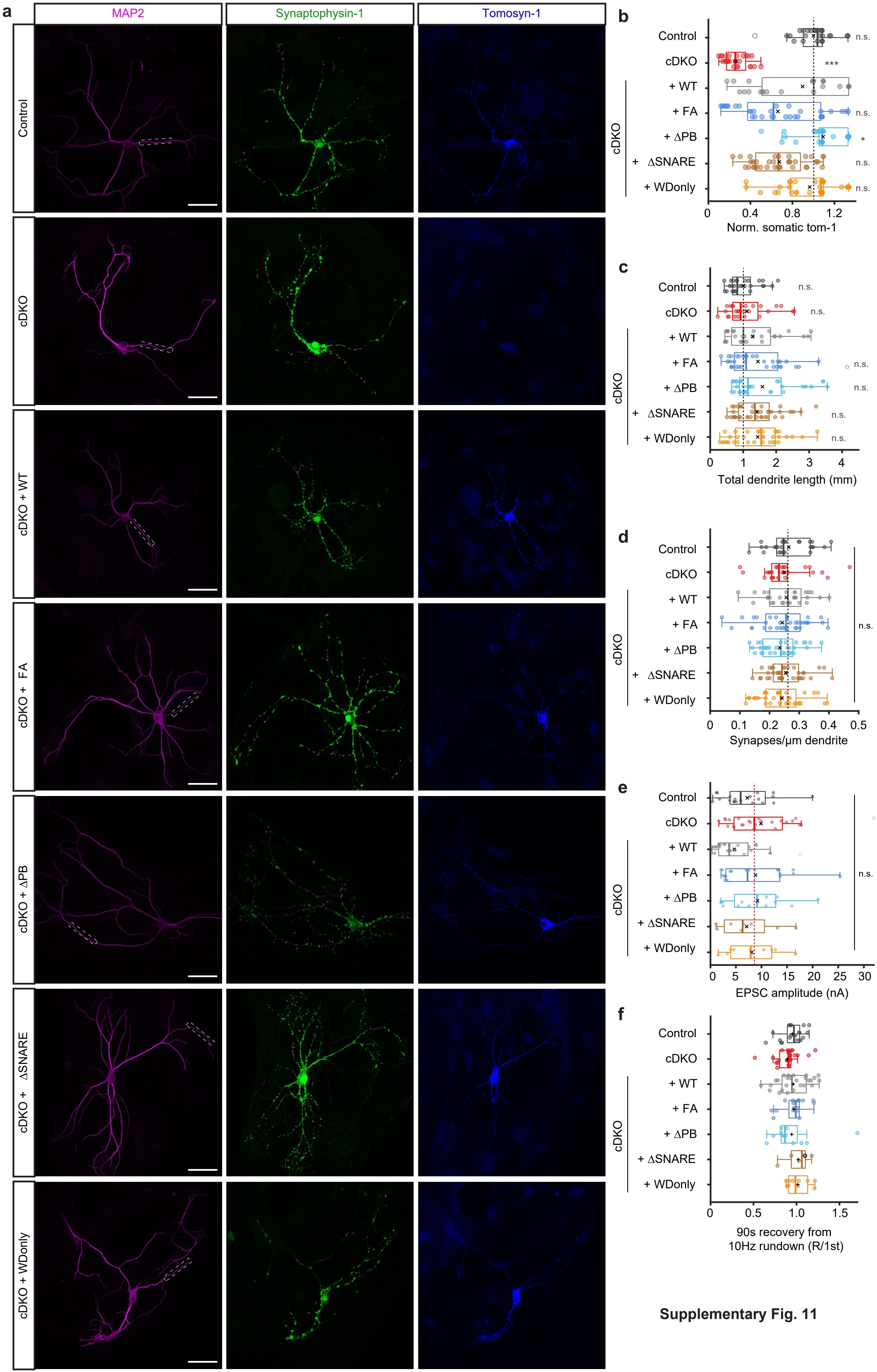

Supplementary Fig. 11

Supplementary Figure 11. **SNARE-truncating constructs support neuronal morphology. a-d** Morphological analysis of neurons infected with different mutant constructs (see Fig. 5). Control n = 26 – 27/3, cDKO n = 27/3, + WT n = 29/3, + FA n = 33 – 34/3, + ΔPB n = 30 – 31/3, + ΔSNARE n = 36 – 38/3, + WDonly n = 34/3. **a** Example images of autaptic hippocampal neurons immunostained for MAP2, synaptophysin-1, and tomosyn-1. Dashed boxes correspond to dendrite zoom-ins in Fig. 6 f. Scale bar = 50 µm. **b** Somatic intensity of tomosyn-1 normalized to controls. Control n = 26/3, cDKO n = 27/3, + WT n = 29/3, + FA n = 33/3, + ΔPB n = 30/3, + ΔSNARE n = 36/3, + WDonly n = 34/3. Post hoc comparisons against +WT: *p*=0.1271 (control); ****p*<0.0001 (cDKO), *p*=0.00927 (+FA), **p*=0.00492 (+ΔPB), *p*=0.009623 (+ΔSNARE), *p*=0.1967 (+WDonly). **c** Total dendrite length as derived from the MAP2 mask. Control n = 27/3, cDKO n = 27/3, + WT n = 29/3, + FA n = 34/3, + ΔPB n = 31/3, + ΔSNARE n = 38/3, + WDonly n = 34/3. Post hoc comparisons against +WT: *p*=0.0567 (control); *p*=0.2442 (cDKO), *p*=0.6569 (+FA), *p*=0.1724 (+ΔPB), *p*=0.2624 (+ΔSNARE), *p*=0.2592 (+WDonly). **d** Synapse density as derived from the synaptophysin-1-positive puncta within the MAP2 mask. Control n = 27/3, cDKO n = 27/3, + WT n = 29/3, + FA n = 34/3, + ΔPB n = 31/3, + ΔSNARE n = 38/3, + WDonly n = 34/3; *p*=0.7383 (all groups). **e** Absolute EPSC amplitudes. Control n = 21/6, cDKO n = 25/8, +WT n = 27/6, +FA n = 18/5, +ΔPB n = 13/4, +ΔSNARE n = 8/4, +WDonly n = 10/3; *p*=0.06294 (all groups). **f** EPSC amplitude measured 90 seconds after STP protocol (5 pulses at 10Hz) normalized to initial EPSC amplitude (1st pulse 10Hz train). Control n = 21/6, cDKO n = 23/8, +WT n = 26/6, +FA n = 16/5, +ΔPB n = 12/4, +ΔSNARE n = 6/3, +WDonly n = 10/3; *p*=0.06294 (all groups).

N = cells/independent cultures. In **b-f**, boxplots display median (center), upper and lower quartiles (box bounds) and whiskers to the last datapoint within 1.5x interquartile range. A one-way ANOVA tested the significance of adding experimental group as a predictor, see Supplementary Table 1. For post-hoc comparison, p-value thresholds (*<0.05; **<0.01;***<0.001) were adjusted with a Bonferroni correction (α/number of tests). Abbreviations: n.s. (not significant). Source data are provided as a Source Data file.

Supplementary Fig. 12

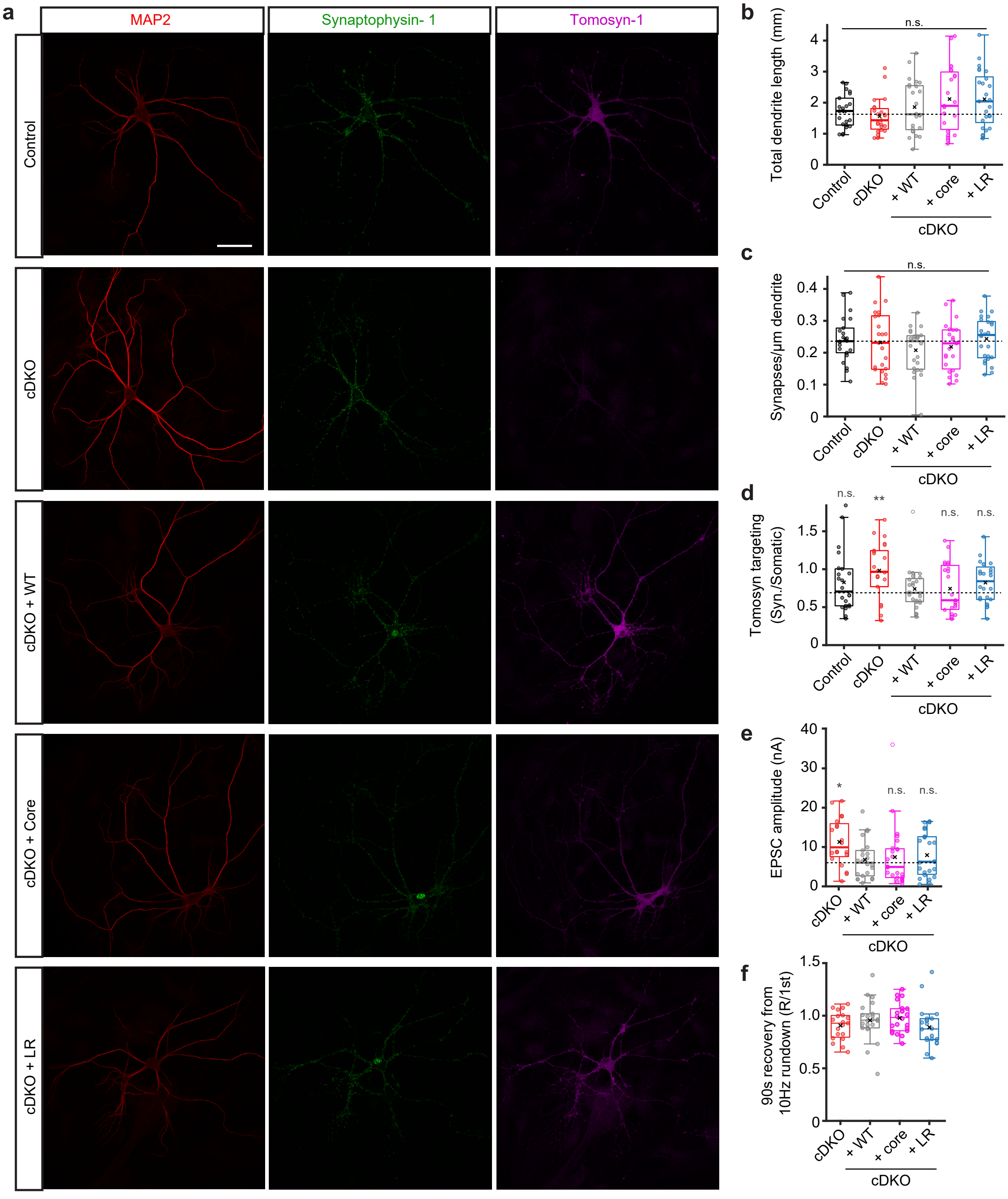

Supplementary Figure 12. **The partial SNARE-hybrid constructs support neuronal morphology. a-d** Morphological analysis of neurons infected with different mutant constructs (see Fig. 7). **a** Example images of autaptic hippocampal neurons immunostained for MAP2, synaptophysin-1, and tomosyn-1. Scale bar = 50 µm. Control n = 22/4, cDKO n = 23/4, + WT n = 24/4, + Core n = 23/4, + LR n = 25/4. **b** Total dendrite length as derived from the MAP2 mask; *p*=0.0553 (all groups). **c** Synapse density as derived from the synaptophysin-1-positive puncta within the MAP2 mask; *p*=0.4820 (all groups). **d** The ratio of the tomosyn-1 signal intensity in synapses to the signal in the soma was calculated to demonstrate synaptic targeting; *p*=0.013 (all groups). Post hoc comparisons against +WT: *p*=0.1836 (control); ***p*=0.00025 (cDKO), *p*=0.6171 (+Core), *p*=0.03678 (+LR). **e** Absolute EPSC amplitudes cDKO. n = 20/4, + WT n = 22/4, + Core n = 24/4, + LR n = 23/4; *p*=0.0054. Post hoc comparisons against +WT: **p*=0.0051 (cDKO), *p*=0.9399 (+Core), *p*=0.3096 (+LR). **f** EPSC amplitude measured 90 seconds after STP protocol (5 pulses at 10Hz) normalized to initial EPSC amplitude (1st pulse 10Hz train). cDKO n = 19/4, + WT n = 22/4, + Core n = 22/4, + LR n = 22/4.

N = cells/independent cultures. In **b-f**, boxplots display median (center), upper and lower quartiles (box bounds) and whiskers to the last datapoint within 1.5x interquartile range. A one-way ANOVA tested the significance of adding experimental group as a predictor, see Supplementary Table 1. For post-hoc comparison, p-value thresholds (*<0.05; **<0.01;***<0.001) were adjusted with a Bonferroni correction (α/number of tests). Abbreviations: n.s. (not significant). Source data are provided as a Source Data file.

PARAMETER	GROUP	(N/n)	MEAN±SD	OUTLIER	ANOVA BETWEEN MODELS	POSTHOC ANOVA ON SUBSETS
SUPPLEMENTAL FIGURE 1						
Synaptic tom-1 intensity (a.u.)	Control	2/31	1399 ± 334	0	F(1,58) = 231.1, p<0.0001 ***	
	cDKO	2/30	424 ± 129	0		
Dendrite length (mm)	Control	4/62	1764 ± 944	0	F(1,114) = 1.107, p =0.295	
	cDKO	4/57	1605 ± 889	0		
Synapses/ µm	Control	4/62	0.27 ± 0.1	1	F(1,113) = 1.636, p=0.2035	
	cDKO	4/57	0.28 ± 0.09	0		
Neurite length (mm), DIV7	Control	1/13	1048 ± 569	0	F(3,60) = 11.54, p<0.0001 ***	WT-DIV7 vs cDKO-DIV7: F(1,25) = 0.0931, p= 0.7628
	cDKO	1/14	1110 ± 471	0		WT-DIV7 vs WT-DIV15: F(1,30) = 18.26, p= 0.0002***
Neurite length (mm), DIV15	Control	1/19	2432 ± 1065	0		WT-DIV15 vs cDKO-DIV15: F(1,35) = 0.8302, p= 0.3685
	cDKO	1/18	2136 ± 899	0		cDKO-DIV7 vs cDKO-DIV15: F(1,30) = 14.96, p= 0.0005**
Synapses/µm, DIV7	Control	1/13	0.11 ± 0.05	0	F(3,59) = 55.81, p<0.0001 ***	WT-DIV7 vs cDKO-DIV7:F(1,25) = 0.0208, p=0.8865
	cDKO	1/14	0.11 ± 0.05	0		WT-DIV7 vs WT-DIV15: F(1,29) = 67.93, p<0.0001 ***
Synapses/µm, DIV15	Control	1/19	0.33 ± 0.14	1		WT-DIV15 vs cDKO-DIV15: F(1,34) = 1.17, p= 0.2869
	cDKO	1/18	0.32 ± 0.07	0		cDKO-DIV7 vs cDKO-DIV15: F(1,30) = 100.9, p<0.0001 ***
Intensity Tom, DIV7	Control	1/13	1399 ± 406	0	F(3,58) = 101.7, p<0.0001 ***	WT-DIV7 vs cDKO-DIV7: F(1,24) = 55.84, p<0.0001 ***
	cDKO	1/14	624 ± 352	1		WT-DIV7 vs WT-DIV15: F(1,29) = 1.806, p= 0.1895
Intensity Tom, DIV15	Control	1/19	1499 ± 268	1		WT-DIV15 vs cDKO-DIV15: F(1,34) = 381, p<0.0001 ***
	cDKO	1/18	412 ± 161	0		cDKO-DIV7 vs cDKO-DIV15: F(1,29) = 5.912, p= 0.0215
FIGURE 1 + SUPPLEMENTAL FIGURE 2						
mEPSC frequency	Control	6/47	6.54 ± 6.54	1	F(1,83) = 111.7, p<0.0001 ***	
	cDKO	6/44	27.11 ± 13.87	0		
mEPSC amplitude	Control	6/47	31.16 ± 5.85	1	F(1,83) = 3.075, p=0.0832	
	cDKO	6/44	32.84 ± 5.68	0		
EPSC amplitude (nA)	Control	6/50	8.4 ± 6.8	1	F(1,91) = 16.44, p=0.0001 ***	
	cDKO	6/49	12.2 ± 5.3	0		
EPSC charge (pC)	Control	6/50	124 ± 145	1	F(1,90) = 14.63, p=0.0002 ***	
	cDKO	6/49	194 ± 121	1		
EPSC rise time (ms)	Control	6/44	1.68 ± 0.64	2	F(1,83) = 0.137, p=0.7124	
	cDKO	6/49	0.64 ± 0.47	1		
EPSC decay time (ms)	Control	6/44	6.92 ± 2.81	1	F(1,85) = 3.627, p=0.0602	
	cDKO	6/49	7.67 ± 2.56	0		
EPSC half-width (ms)	Control	6/44	9.46 ± 2.96	1	F(1,83) = 2.929, p=0.0907	
	cDKO	6/49	10.22 ± 3.06	0		
PAIRED PULSE RATIO (#2/#1)						
20 ms IPI	Control	6/45	0.95 ± 0.35	1	F(1,83) = 17.25, p<0.0001 ***	
	cDKO	6/47	0.71 ± 0.23	1		
50 ms IPI	Control	6/37	1.11 ± 0.41	1	F(1,70) = 27.70, p<0.0001***	
	cDKO	6/42	0.74 ± 0.20	1		
100 ms IPI	Control	6/39	1.04 ± 0.33	0	F(1,76) = 22.77, p<0.0001***	
	cDKO	6/44	0.75 ± 0.17	0		
200 ms IPI	Control	6/47	0.95 ± 0.19	1	F(1,86) = 37.86, p<0.0001***	
	cDKO	6/47	0.74 ± 0.14	0		
SHORT-TERM PLASTICITY (#5/#1)						
5 Hz train	Control	6/47	0.90 ± 0.36	1	F(1,86) = 69.15, p<0.0001***	
	cDKO	6/47	0.50 ± 0.15	0		
10 Hz train	Control	6/39	0.85 ± 0.43	1	F(1,75) = 32.54, p<0.0001***	
	cDKO	6/44	0.44 ± 0.16	0		
20 Hz train	Control	6/39	0.74 ± 0.37	1	F(1,70) = 35.16, p<0.0001***	
	cDKO	6/42	0.38 ± 0.20	1		
SUPPLEMENTAL FIGURE 3						
mEPSC frequency	Control	4/21	5.35 ± 6.77	1	F(1,34) = 51.05, p<0.0001 ***	
	cDKO	4/21	37.39 ± 19.02	0		
mEPSC amplitude	Control	4/21	20.55 ± 8.83	1	F(1,33) = 1.21, p=0.2802	
	cDKO	4/21	24.41 ± 15.39	1		
EPSC amplitude	Control	5/23	8.73 ± 4.25	0	F(1,40) = 2.19, p=0.1463	
	cDKO	5/23	10.82 ± 5.67	0		
5Hz STP (#5/#1)	Control	5/23	0.62 ± 0.17	1	F(1,39) = 30.60, p<0.0001 ***	
	cDKO	5/23	0.40 ± 0.10	0		
SUPPLEMENTAL FIGURE 4						
PPR (#2/#1), 50 ms IPI						
1mM $[Ca^{2+}]e$	Control	4/13	1.57 ± 0.54	0	F(1,22) = 9.620, p= 0.0052**	
	cDKO	4/14	1.12 ± 0.14	0		
1.5mM $[Ca^{2+}]e$	Control	4/9	1.18 ± 0.33	0	F(1,18) = 8.703, p=0.0086**	
	cDKO	4/14	0.85 ± 0.16	0		
2mM $[Ca^{2+}]e$	Control	4/13	0.98 ± 0.20	0	F(1,22) = 20.34, p= 0.0002***	
	cDKO	4/14	0.72 ± 0.08	0		
4mM $[Ca^{2+}]e$	Control	4/9	0.74 ± 0.14	0	F(1,15) = 12.79, p=0.0028**	
	cDKO	4/11	0.56 ± 0.07	0		
8mM $[Ca^{2+}]e$	Control	4/9	0.63 ± 0.1	0	F(1,14) = 5.932, p=0.0288*	
	cDKO	4/10	0.51 ± 0.08	0		
Normalized EPSC amp. (to 2mM)						
KD Ca^{2+} (mM)	Control	4/9	1.61 ± 0.35	0	F(1,15) = 6.865, p= 0.0193*	
	cDKO	4/11	1.27 ± 0.2	0		
Hill coefficient (*c*)	Control	4/9	3.02 ± 0.57	0	F(1,15) = 0.082, p=0.7792	
	cDKO	4/11	2.87 ± 0.45	0		
FIGURE 2 + SUPPLEMENTAL FIGURE 5						
RRP size (nC) - manual	Control	6/25	2.45 ± 1.26	0	F(1,43) = 1.778, p=0.1894	
	cDKO	6/26	2.15 ± 1.59	0		
Pves (EPSC/RRP) - manual	Control	6/24	0.04 ± 0.03	0	F(1,42) = 6.626, p=0.0137*	
	cDKO	6/25	0.07 ± 0.06	0		
250/500mM - manual	Control	6/25	0.45 ± 0.21	0	F(1,43) = 42.13, p<0.0001***	
	cDKO	6/26	0.85 ± 0.24	0		
Refilled pool (% of initial)	Control	6/19	0.79 ± 0.07	0	F(1,32) = 0.230, p= 0.6347	
	cDKO	6/20	0.80 ± 0.09	0		
RRP size (nC) – fitted	Control	6/16	1.99 ± 1.02	0	F(1,27) = 0.687, p=0.4144	
	cDKO	6/18	1.33 ± 0.91	0		
Pves (EPSC/RRP) – fitted	Control	6/15	0.05 ± 0.04	0	F(1,23) = 2.080, p=0.1627	
	cDKO	6/15	0.10 ± 0.06	0		
250/500mM - fitted	Control	6/10	0.55 ± 0.09	0	F(1,19) = 129.3, p<0.0001***	

	cDKO	6/15	0.92 ± 0.06	0		
$K_{2,max}(s^{-1})$						
0mM HS	Control	6/13	0.0001 ± 0.00008	0	F(1,14) = 6.366, p= 0.02436*	
	cDKO	6/7	0.0013 ± 0.0010	0		
250mM HS	Control	6/12	0.43 ± 0.81	0	F(1,22) = 21.34, p=0.0001***	
	cDKO	6/17	0.80 ± 0.42	1		
500mM HS	Control	6/16	3.02 ± 3.53	0	F(1,26) = 41.96, p<0.0001***	
	cDKO	6/18	5.13 ± 1.19	0		
ΔE_a(RT)						
0mM HS	Control	6/13	0.00 ± 0.98	0	F(1,14) = 21.03, p= 0.0004***	
	cDKO	6/7	-2.43 ± 0.68	0		
250mM HS	Control	6/12	-7.84 ± 0.85	0	F(1,23) = 21.36, p= 0.0001***	
	cDKO	6/17	-8.95 ± 0.44	0		
500mM HS	Control	6/16	-10.1 ± 0.63	0	F(1,26) = 48.63, p< 0.0001***	
	cDKO	6/18	-10.9 ± 0.24	1		
Priming rate $K_1(s^{-1})$	Control	6/16	0.17 ± 0.20	1	F(1,24) = 3.768, p=0.06409	
	cDKO	6/16	0.07 ± 0.14	0		
Unpriming rate $K_{-1}(s^{-1})$	Control	6/16	0.07 ± 0.07	1	F(1,24) = 3.959, p=0.05814	
	cDKO	6/16	0.04 ± 0.06	0		
SYNAPTIC RECOVERY AFTER TRAIN STIMULATION						
40Hz train						
Norm. EPSC – R1	Control	6/33	1.00 ± 0.31	0	F(1,64) = 53.49, p<0.0001***	
	cDKO	6/38	0.59 ± 0.13	0		
Norm. EPSC – R2	Control	6/31	0.95 ± 0.21	0	F(1,59) = 1.732, p=0.1933	
	cDKO	6/35	0.90 ± 0.14	0		
1st EPSC (nA)	Control	6/33	6.95 ± 4.85	1	F(1,62) = 7.586, p<0.007711**	
	cDKO	6/38	9.51 ± 5.08	1		
R1 EPSC (nA)	Control	6/33	6.38 ± 4.42	1	F(1,62) = 0.485, p=0.4889	
	cDKO	6/38	5.57 ± 2.94	1		
R2 EPSC (nA)	Control	6/31	6.36 ± 4.89	1	F(1,57) = 4.858, p=0.03157*	
	cDKO	6/35	8.14 ± 4.54	1		
RRP size (nC)	Control	6/33	0.78 ± 0.51	0	F(1,63) = 2.642, p=0.109	
	cDKO	6/38	0.61 ± 0.46	1		
Refilling rate (nC/s)	Control	6/33	1.05 ± 1.00	1	F(1,61) = 2.214, p=0.1419	
	cDKO	6/38	0.83 ± 0.61	2		
FITTED PARAMETERS RECOVERY 500mM HS						
$K_{2,max}(s^{-1})$	Control	6/12	2.06 ± 1.31	0	F(1,16) = 0.738, p= 0.403	
	cDKO	6/11	2.52 ± 1.10	0		
$\Delta E_a(RT)$	Control	6/12	-9.83 ± 0.64	0	F(1,16) = 0.691, p=0.4182	
	cDKO	6/11	-10.03 ± 0.72	0		
SUPPLEMENTAL FIGURE 6						
Micro-network Docked SVs (µm)	Control	4/132	8.11 ± 3.78	2	L ratio (2,339) = 7.153, p=0.2044	*Control-cDKO:* p=0.0424
	cDKO	4/89	6.57 ± 3.45	1		*Control-WT:* p=0.0153*
	+WT	4/128	6.87 ± 4.57	2		*cDKO-WT:* p=0.804
Micro-network AZ length (µm)	Control	4/132	623 ± 271	2	L ratio (2,335) = 3.176, p=0.2044	
	cDKO	4/89	580 ± 308	4		
	+WT	4/128	643 ± 285	3		
Micro-network Total SV number	Control	4/132	154 ± 96	4	L ratio (2,335) = 2.344, p=0.3097	
	cDKO	4/89	134 ± 92	3		
	+WT	4/128	143 ± 99	2		
Micro-network Contacting SVs outside AZ	Control	4/132	0.42 ± 1.04	2	L ratio (2, 339) = 3.118, p=0.2103	
	cDKO	4/89	0.20 ± 0.65	1		
	+WT	4/128	0.16 ± 0.57	2		
FIGURE 3 + SUPPLEMENTAL FIGURE 7						
EPSC amp. (nA)	cDKO	4/17	9.73 ± 5.33	0	F(2,52) = 7.508, p=0.001366**	*vsWT:* F(1,32) = 12.30, p=0.001366**
	+WT	4/21	5.03 ± 6.13	1		
	+Hybrid	4/21	10.75 ± 7.33	0		*vsWT:* F(1,36) = 11.87, p=0.001468**
STP (5th/1st)	cDKO	4/17	0.35 ± 0.15	0	F(2,53) = 15.08, p<0.0001***	*vsWT:* F(1,33) = 25.50, p<0.0001***
	+WT	4/21	0.93 ± 0.47	0		
	+Hybrid	4/21	0.54 ± 0.27	0		*vsWT:* F(1,37) = 10.76, p=0.002262**
EPSC recovery	cDKO	4/14	0.51 ± 0.10	0	F(2,46) = 28.71, p<0.0001***	*vsWT:* F(1,29) = 36.20, p<0.0001***
	+WT	4/20	1.48 ± 0.67	0		
	+Hybrid	4/18	0.65 ± 0.23	0		*vsWT:* F(1,33) = 30.10, p<0.0001***
Norm. synaptic tom-1	Control	3/35	1.00 ± 0.26	0	F(3,125) = 50.52, p<0.0001***	*vsWT:* F(1,63) = 0.749, p=0.3902
	cDKO	3/30	0.35 ± 0.16	0		*vsWT:* F(1,58) = 50.52, p<0.0001***
	+WT	3/33	1.12 ± 0.48	1		
	+hybrid	3/35	1.69 ± 0.88	1		*vsWT:* F(1,62) = 15.89, p=0.0001797***
Synaptic / somatic tomosyn	Control	3/35	0.66 ± 0.40	0	F(3,124) = 22.94, p<0.0001***	*vsWT:* F(1,63) = 1.123, p=0.2934
	cDKO	3/30	1.04 ± 0.44	0		*vsWT:* F(1,58) = 45.35, p<0.0001***
	+WT	3/33	0.60 ± 0.25	1		
	+hybrid	3/35	0.65 ± 0.27	1		*vsWT:* F(1,63) = 0.805, p=0.373
Total dendrite length (mm)	Control	3/35	2.36 ± 1.07	0	F(3,127) = 6.009, p=0.0007***	*vsWT:* F(1,64) = 3.602, p=0.06222
	cDKO	3/30	1.72 ± 0.69	0		*vsWT:* F(1,59) = 3.118, p= 0.0826
	+WT	3/33	2.05 ± 0.87	0		
	+hybrid	3/35	2.45 ± 1.17	0		*vsWT:* F(1,64) = 4.617, p= 0.03544
Synapses/µm dendrite	Control	3/35	0.28 ± 0.09	0	F(3,127) = 1.237, p= 0.2991	
	cDKO	3/30	0.26 ± 0.08	0		
	+WT	3/33	0.30 ± 0.09	0		
	+hybrid	3/35	0.30 ± 0.10	0		
FIGURE 7 + SUPPLEMENTAL FIGURE 11						
EPSC amp. (nA)	Control	6/21	7.23 ± 4.90	0	F(6,106) = 2.069, p=0.06294	
	cDKO	8/25	9.89 ± 6.77	1		
	+WT	6/27	4.69 ± 4.22	1		
	+FA	5/18	8.81 ± 6.61	0		
	+ΔPB	4/13	9.20 ± 5.68	0		
	+ΔSNARE	4/8	7.08 ± 5.46	0		
	+WDonly	3/10	8.07 ± 4.83	0		
STP (5th/1st)	Control	6/21	0.78 ± 0.41	0	F(6,108) = 13.21, p<0.0001 ***	*vsKO:* F(1,37) = 19.35, p=0.00011***
	cDKO	8/25	0.44 ± 0.16	0		
	+WT	6/27	1.25 ± 0.72	0		*vsKO:* F(1,43) = 26.30, p<0.0001***
	+FA	5/18	0.50 ± 0.14	0		*vsKO:* F(1,34) = 1.102, p=0.3013
	+ΔPB	4/13	0.37 ± 0.08	0		*vsKO:* F(1,29) = 0.024, p= 0.8781

	+ΔSNARE	4/8	0.44 ± 0.20	0		*vsKO:* F(1,24) = 0.078, p= 0.7821
	+WDonly	3/10	0.41 ± 0.16	0		*vsKO:* F(1,26) = 1.331, p=0.2592
EPSC recovery	Control	6/21	0.94 ± 0.36	0	F(6,96) = 16.18, p<0.0001 ***	*vsKO:* F(1,34) = 30.28, p=0.00011***
	cDKO	8/22	0.53 ± 0.16	0		
	+WT	6/25	1.49 ± 0.79	1		*vsKO:* F(1,37) = 41.17, p<0.0001***
	+FA	5/16	0.69 ± 0.24	0		*vsKO:* F(1,29) = 4.793, p=0.03677
	+ΔPB	4/12	0.52 ± 0.15	0		*vsKO:* F(1,25) = 0.524, p=0.4757
	+ΔSNARE	3/6	0.56 ± 0.15	0		*vsKO:* F(1,19) = 0.173, p=0.682
	+WDonly	2/9	0.52 ± 0.14	0		*vsKO:* F(1,22) = 0.036, p=0.8506
Norm. synaptic tom-1 levels	Control	3/27	1.00 ± 0.29	1	F(6,206) = 25.82, p<0.0001 ***	*vsWT:* F(1,51) = 1.909, p=0.1731; *vsKO:* F(1,49) = 285, p<0.0001***
	cDKO	3/27	0.22 ± 0.05	0		
	+WT	3/29	1.13 ± 0.85	0		*vsKO:* F(1,52) = 48.47, p<0.0001***
	+FA	3/34	0.56 ± 0.40	1		*vsWT:* F(1,58) = 19.22, p<0.0001***; *vsKO:* F(1,56) = 33.43, p<0.0001***
	+ΔPB	3/31	1.48 ± 1.03	0		*vsWT:* F(1,56) = 4.163, p=0.04605; *vsKO:* F(1,54) = 53.70, p<0.0001***
	+ΔSNARE	3/38	0.42 ± 0.21	1		*vsWT:* F(1,61) = 29.97, p<0.0001***; *vsKO:* F(1,59) = 51.26, p<0.0001***
	+WDonly	3/34	0.64 ± 0.30	1		*vsWT:* F(1,58) = 13.40, p= 0.00054**; *vsKO:* F(1,56) = 89.86, p<0.0001***
Norm. somatic tom-1 levels	Control	3/26	1.00 ± 0.18	1	F(6,205) = 34.03, p<0.0001 ***	*vsWT:* F(1,50) = 2.408, p=0.1271
	cDKO	3/27	0.26 ± 0.11	0		vsWT: F(1,52) = 78.04, p<0.0001***
	+WT	3/29	0.90 ± 0.41	0		
	+FA	3/33	0.66 ± 0.38	0		*vsWT:* F(1,58) = 7.25, p=0.009266
	+ΔPB	3/30	1.09 ± 0.22	0		*vsWT:* F(1,55) = 8.59, p= 0.004917*
	+ΔSNARE	3/36	0.68 ± 0.25	0		*vsWT:* F(1,61) = 7.147, p=0.009623
	+WDonly	3/34	0.96 ± 0.27	0		*vsWT:* F(1,59) = 1.705, p= 0.1967
Total dendritic length (mm)	Control	3/27	1.00 ± 0.45	0	F(6,210) = 2.871, p= 0.0104*	*vsWT:* F(1,52) = 3.798, p= 0.05672
	cDKO	3/27	1.11 ± 0.62	0		*vsWT:* F(1,52) = 1.387, p= 0.2442
	+WT	3/29	1.29 ± 0.79	0		
	+FA	3/34	1.45 ± 0.89	1		*vsWT:* F(1,58) = 0.199, p= 0.6569
	+ΔPB	3/31	1.58 ± 0.93	0		*vsWT:* F(1,56) = 1.911, p= 0.1724
	+ΔSNARE	3/38	1.42 ± 0.66	0		*vsWT:* F(1,63) = 1.279, p= 0.2624
	+WDonly	3/34	1.44 ± 0.75	0		*vsWT:* F(1,59) = 1.298, p= 0.2592
Synapses/µm dendrite	Control	3/27	0.26 ± 0.07	0	F(6,211) = 0.590, p= 0.7383	
	cDKO	3/27	0.25 ± 0.08	0		
	+WT	3/29	0.26 ± 0.07	0		
	+FA	3/34	0.24 ± 0.09	0		
	+ΔPB	3/31	0.23 ± 0.06	0		
	+ΔSNARE	3/38	0.25 ± 0.06	0		
	+WDonly	3/34	0.24 ± 0.07	0		
FIGURE 8 + SUPPLEMENTAL FIGURE 12						
EPSC amp. (nA)	cDKO	4/20	11.30 ± 5.99	0	F(3,81) = 4.542, p=0.005406**	*vsWT:* F(1,37) = 8.863, p=0.005108*
	+WT	4/22	6.78 ± 4.89	0		
	+Core	4/24	7.44 ± 7.74	1		*vsWT:* F(1,40) = 0.006, p=0.9399
	+LR	4/23	7.95 ± 5.59	0		*vsWT:* F(1,40) = 1.059, p=0.3096
STP ($5^{th}/1^{st}$)	cDKO	4/20	0.35 ± 0.09	0	F(3,81) = 28.33, p<0.0001***	*vsWT:* F(1,37) = 52.42, p<0.0001***
	+WT	4/22	0.97 ± 0.44	0		
	+Core	4/24	0.56 ± 0.17	0		*vsWT:* F(1,41) = 34.32, p<0.0001***
	+LR	4/23	0.58 ± 0.41	1		*vsWT:* F(1,39) = 29.40, p<0.0001***
EPSC recovery	cDKO	4/19	0.54 ± 0.11	0	F(3,77) = 15.46, p<0.0001***	*vsWT:* F(1,35) = 36.88, p<0.0001***
	+WT	4/22	1.52 ± 0.98	1		
	+Core	4/22	1.00 ± 0.62	0		*vsWT:* F(1,38) = 9.200, p=0.004347*
	+LR	4/22	0.79 ± 0.33	0		*vsWT:* F(1,38) = 21.07, p<0.0001***
Norm. synaptic tom-1 levels	Control	4/22	1.00 ± 0.28	0	F(4,109) = 16.67, p<0.0001***	*vsWT:* F(1,41) = 0.099, p=0.7549
	cDKO	4/23	0.30 ± 0.12	0		*vsWT:* F(1,42) = 57.04, p<0.0001***
	+WT	4/24	1.04 ± 0.52	0		
	+Core	4/23	0.91 ± 0.58	0		*vsWT:* F(1,42) = 0.936, p=0.3389
	+LR	4/25	1.21 ± 0.53	0		*vsWT:* F(1,44) = 0.923, p=0.3419
Synaptic / somatic tomosyn	Control	4/22	0.83 ± 0.40	0	F(4,106) = 3.332, p=0.013*	*vsWT:* F(1,40) = 1.831, p=0.1836
	cDKO	4/23	0.98 ± 0.37	0		*vsWT:* F(1,40) = 16.13, p=0.0002531**
	+WT	4/24	0.74 ± 0.28	1		
	+Core	4/23	0.74 ± 0.33	0		*vsWT:* F(1,41) = 0.254, p=0.6171
	+LR	4/25	0.82 ± 0.27	0		*vsWT:* F(1,42) = 4.653, p=0.03678
Total dendritic length (mm)	Control	4/22	1.72 ± 0.53	0	F(4,109) = 2.389, p=0.0553	*vsWT:* F(1,41) = 0.430, p=0.5159
	cDKO	4/23	1.56 ± 0.57	0		*vsWT:* F(1,42) = 2.220, p= 0.1437
	+WT	4/24	1.86 ± 0.85	0		
	+Core	4/23	2.12 ± 1.05	0		*vsWT:* F(1,42) = 1.493, p= 0.2286
	+LR	4/25	2.11 ± 0.91	0		*vsWT:* F(1,44) = 0.911, p= 0.3451
Synapses/µm dendrite	Control	4/22	0.24 ± 0.07	0	F(4,109) = 0.874, p= 0.482	
	cDKO	4/23	0.23 ± 0.10	0		
	+WT	4/24	0.21 ± 0.07	0		
	+Core	4/23	0.22 ± 0.07	0		
	+LR	4/25	0.24 ± 0.07	0		

Supplementary table 1. **Overview of statistical analyses per figure.** The results of the one-way ANOVA to test whether the more complex model (including information on the experimental group observations belong to) is significantly better at capturing the data than the simpler model. Results are reported as F(between groups df, within groups df) = [F-value], p = [p-value]. P-value thresholds = *<0.05; **<0.01;***<0.001. For post-hoc tests, p-value thresholds were adjusted with a Bonferroni correction (α/number of tests). N = independent cultures, n = neurons.
