# Supplementary material for: Tomosyns attenuate SNARE assembly and synaptic depression by binding to VAMP2-containing template complexes
Source: Nat Commun. 2024 Mar 26;15:2652. doi: 10.1038/s41467-024-46828-1 (PMC10965968; doi:10.1038/s41467-024-46828-1)
Supplement: Supplementary file 3 — Reporting Summary [file 41467_2024_46828_MOESM3_ESM.pdf]

nature portfolio

Corresponding author(s): M. Meijer, J.Yang, Y. Zhang and M.Verhage

Last updated by author(s): Mar 8, 2024

Reporting Summary

Nature Portfolio wishes to improve the reproducibility of the work that we publish. This form provides structure for consistency and transparency in reporting. For further information on Nature Portfolio policies, see our Editorial Policies and the Editorial Policy Checklist.

Statistics

For all statistical analyses, confirm that the following items are present in the figure legend, table legend, main text, or Methods section.

n/a	Confirmed	
☐	☒	The exact sample size (*n*) for each experimental group/condition, given as a discrete number and unit of measurement
☐	☒	A statement on whether measurements were taken from distinct samples or whether the same sample was measured repeatedly
☐	☒	The statistical test(s) used AND whether they are one- or two-sided<br>*Only common tests should be described solely by name; describe more complex techniques in the Methods section.*
☐	☒	A description of all covariates tested
☐	☒	A description of any assumptions or corrections, such as tests of normality and adjustment for multiple comparisons
☐	☒	A full description of the statistical parameters including central tendency (e.g. means) or other basic estimates (e.g. regression coefficient) AND variation (e.g. standard deviation) or associated estimates of uncertainty (e.g. confidence intervals)
☐	☒	For null hypothesis testing, the test statistic (e.g. *F*, *t*, *r*) with confidence intervals, effect sizes, degrees of freedom and *P* value noted<br>*Give P values as exact values whenever suitable.*
☒	☐	For Bayesian analysis, information on the choice of priors and Markov chain Monte Carlo settings
☐	☒	For hierarchical and complex designs, identification of the appropriate level for tests and full reporting of outcomes
☒	☐	Estimates of effect sizes (e.g. Cohen's *d*, Pearson's *r*), indicating how they were calculated

Our web collection on statistics for biologists contains articles on many of the points above.

Software and code

Policy information about availability of computer code

Data collection	not applicable
Data analysis	Published code from the semi-automatic image analysis platform SynD is available at github.com/Hjorthmedh/SynD. Custom code to analyze electrophysiological data is available at github.com/vhuson/viewEPSC. Citations to our code has been added by linking the Github repositories to Zenodo.

For manuscripts utilizing custom algorithms or software that are central to the research but not yet described in published literature, software must be made available to editors and reviewers. We strongly encourage code deposition in a community repository (e.g. GitHub). See the Nature Portfolio guidelines for submitting code & software for further information.

Data

Policy information about availability of data

All manuscripts must include a data availability statement. This statement should provide the following information, where applicable:

- Accession codes, unique identifiers, or web links for publicly available datasets
- A description of any restrictions on data availability
- For clinical datasets or third party data, please ensure that the statement adheres to our policy

Source data are provided with this paper. Other data and material are available from the corresponding authors upon reasonable request.

Research involving human participants, their data, or biological material

Policy information about studies with human participants or human data. See also policy information about sex, gender (identity/presentation), and sexual orientation and race, ethnicity and racism.

Reporting on sex and gender	*Use the terms sex (biological attribute) and gender (shaped by social and cultural circumstances) carefully in order to avoid confusing both terms. Indicate if findings apply to only one sex or gender; describe whether sex and gender were considered in study design; whether sex and/or gender was determined based on self-reporting or assigned and methods used. Provide in the source data disaggregated sex and gender data, where this information has been collected, and if consent has been obtained for sharing of individual-level data; provide overall numbers in this Reporting Summary. Please state if this information has not been collected. Report sex- and gender-based analyses where performed, justify reasons for lack of sex- and gender-based analysis.*
Reporting on race, ethnicity, or other socially relevant groupings	*Please specify the socially constructed or socially relevant categorization variable(s) used in your manuscript and explain why they were used. Please note that such variables should not be used as proxies for other socially constructed/relevant variables (for example, race or ethnicity should not be used as a proxy for socioeconomic status). Provide clear definitions of the relevant terms used, how they were provided (by the participants/respondents, the researchers, or third parties), and the method(s) used to classify people into the different categories (e.g. self-report, census or administrative data, social media data, etc.) Please provide details about how you controlled for confounding variables in your analyses.*
Population characteristics	*Describe the covariate-relevant population characteristics of the human research participants (e.g. age, genotypic information, past and current diagnosis and treatment categories). If you filled out the behavioural & social sciences study design questions and have nothing to add here, write "See above."*
Recruitment	*Describe how participants were recruited. Outline any potential self-selection bias or other biases that may be present and how these are likely to impact results.*
Ethics oversight	*Identify the organization(s) that approved the study protocol.*

Note that full information on the approval of the study protocol must also be provided in the manuscript.

Field-specific reporting

Please select the one below that is the best fit for your research. If you are not sure, read the appropriate sections before making your selection.

[x] Life sciences [ ] Behavioural & social sciences [ ] Ecological, evolutionary & environmental sciences

For a reference copy of the document with all sections, see nature.com/documents/nr-reporting-summary-flat.pdf

Life sciences study design

All studies must disclose on these points even when the disclosure is negative.

Sample size	Sample sizes are in line with the accepted standards from literature.
Data exclusions	Electrophysiological recordings were excluded if series resistance was higher than 15 Mohm, leak current exceeded 300 pA or EPSC size was below 300pA. GABAergic currents were identified based on their postsynaptic decay kinetics or with pharmacological blockers and excluded. These pre-established exclusion criteria provide a quality check for patch-clamp recordings.
Replication	Measurements are taken from individual synapses/neurons from multiple biological replicates, typically between 4-6 independent neuronal cultures. The initial findings in cDKO neurons (faster depression and slower recovery) were reproduced in four subsequent datasets (Fig. 3, 7, 8 and Supplementary Fig. 3).
Randomization	Neuronal cultures were allocated into experimental groups based on a rotating position in the 12-well culture plates.
Blinding	Investigators were blinded to group allocation during data collection and analysis.

Reporting for specific materials, systems and methods

We require information from authors about some types of materials, experimental systems and methods used in many studies. Here, indicate whether each material, system or method listed is relevant to your study. If you are not sure if a list item applies to your research, read the appropriate section before selecting a response.

Materials & experimental systems

n/a	Involved in the study	
☐	☒	Antibodies
☒	☐	Eukaryotic cell lines
☒	☐	Palaeontology and archaeology
☐	☒	Animals and other organisms
☒	☐	Clinical data
☒	☐	Dual use research of concern
☒	☐	Plants

Methods

n/a	Involved in the study	
☒	☐	ChIP-seq
☒	☐	Flow cytometry
☒	☐	MRI-based neuroimaging

Antibodies

Antibodies used	polyclonal chicken anti-MAP2, Abcam, Cat#ab5392<br>polyclonal guinea pig anti-synaptophysin-1, Synaptic Systems, Cat#101004<br>polyclonal rabbit anti-tomosyn-1, Synaptic Systems, Cat#183103<br>monoclonal mouse anti-VAMP2, Synaptic Systems, Cat. No. 104 211<br>monoclonal mouse anti-SNAP25, Covance, Cat# SMI-81R<br>monoclonal mouse anti-syntaxin, Sigma, Cat# S0664<br>monoclonal mouse anti-actin, Chemicon, Cat# MAB1501
Validation	According to the manufacturer, all used antibodies are validated and suitable for western blot and immunocytochemistry and react with mouse. The following antibodies were KO validated: anti-VAMP2 (SySy) and anti-tomosyn1.

Animals and other research organisms

Policy information about studies involving animals; ARRIVE guidelines recommended for reporting animal research, and Sex and Gender in Research

Laboratory animals	Tom1lox/Tom2lox mice were obtained from mating C57Bl/6 Tom1lox mice (Cyagen Biosciences) with C57Bl/6 Tom2lox mice (Cyagen Biosciences). Each neuronal culture (N) was prepared from a single newborn (P1) pup, and independent cultures were taken from different litters. Information on sex of the P1 pups was not collected.<br>Rat glia were prepared from the cortices of newborn rats (Wistar, strain code 003).
Wild animals	The study did not involve wild animals.
Reporting on sex	Information on the sex of newborn animals was not collected or taken into account in the study design.
Field-collected samples	The study did not involve samples collected from the field.
Ethics oversight	Animal experiments were conducted under a CCD-protocol issued by the Central Authority for Scientific Procedures on Animals (CCD - Centrale Commissie Dierproeven) and approved by the Dutch government (VWA, Netherlands Food and Consumer Product Safety Authority) and local authorities (DEC, IvD VU-VUmc).

Note that full information on the approval of the study protocol must also be provided in the manuscript.
